# Supplementary material for: Glutamine Produces Ammonium to Tune Lysosomal pH and Regulate Lysosomal Function
Source: Cells. 2022 Dec 24;12(1):80. doi: 10.3390/cells12010080 (PMC9819001; doi:10.3390/cells12010080)
Supplement: Supplementary file 1 [file cells-12-00080-s001.zip › cells-2029935-supplementary.pdf]

Supplementary Figures

Glutamine produces ammonium to tune lysosomal pH and regulate lysosomal function

Jian Xiong[1,2], Thi Thu Trang Luu[1,2], Kartik Venkatachalam[1,2,3]; Guangwei Du[1,2], Michael X. Zhu[1,2,3,*]

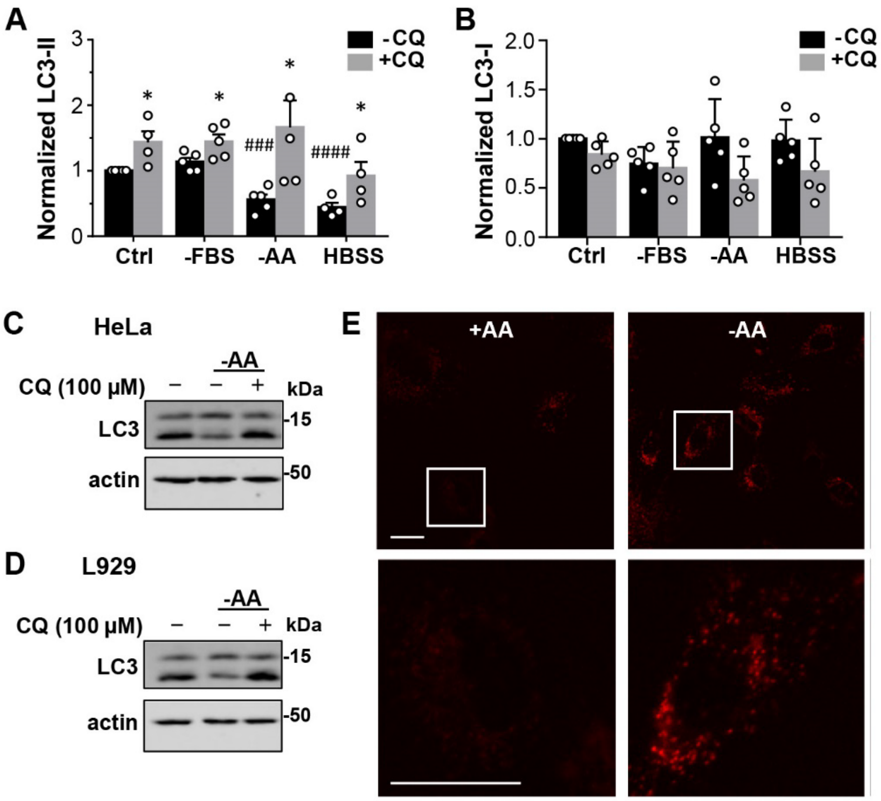

Figure S1. Amino acid starvation accelerates LC3-II degradation in Hela cells and L929 cells.
(**A, B**) Statistics of LC3-II (**A**) and LC3-I (**B**) levels normalized to that of untreated Ctrl for conditions shown in **Fig. 1A** from n = 4-5 experiments; LC3-II/actin and LC3-I/actin ratios were used for normalization. * $p < 0.05$ *vs*. corresponding +CQ by Grouped analyses - multiple *t* tests; $^{###}$ $p < 0.001$, $^{####}$ $p < 0.0001$ *vs*. Ctrl - CQ by one-way ANOVA with Dunnett's multiple comparisons test. (**C, D**) Western blot analysis of LC3 and actin of HeLa (**C**) and L929 (**D**) cells cultured in normal medium or subject to 1-hour amino acid starvation in the absence or presence of CQ (100 μM). (**E**) Same images as that shown in **Fig. 1D**, with the red DQ-BSA fluorescence in black background and single cells in boxed areas enlarged (*lower panels*) to show lysosomes containing the degraded (dequenched) products. Scale bars, 20 μm.

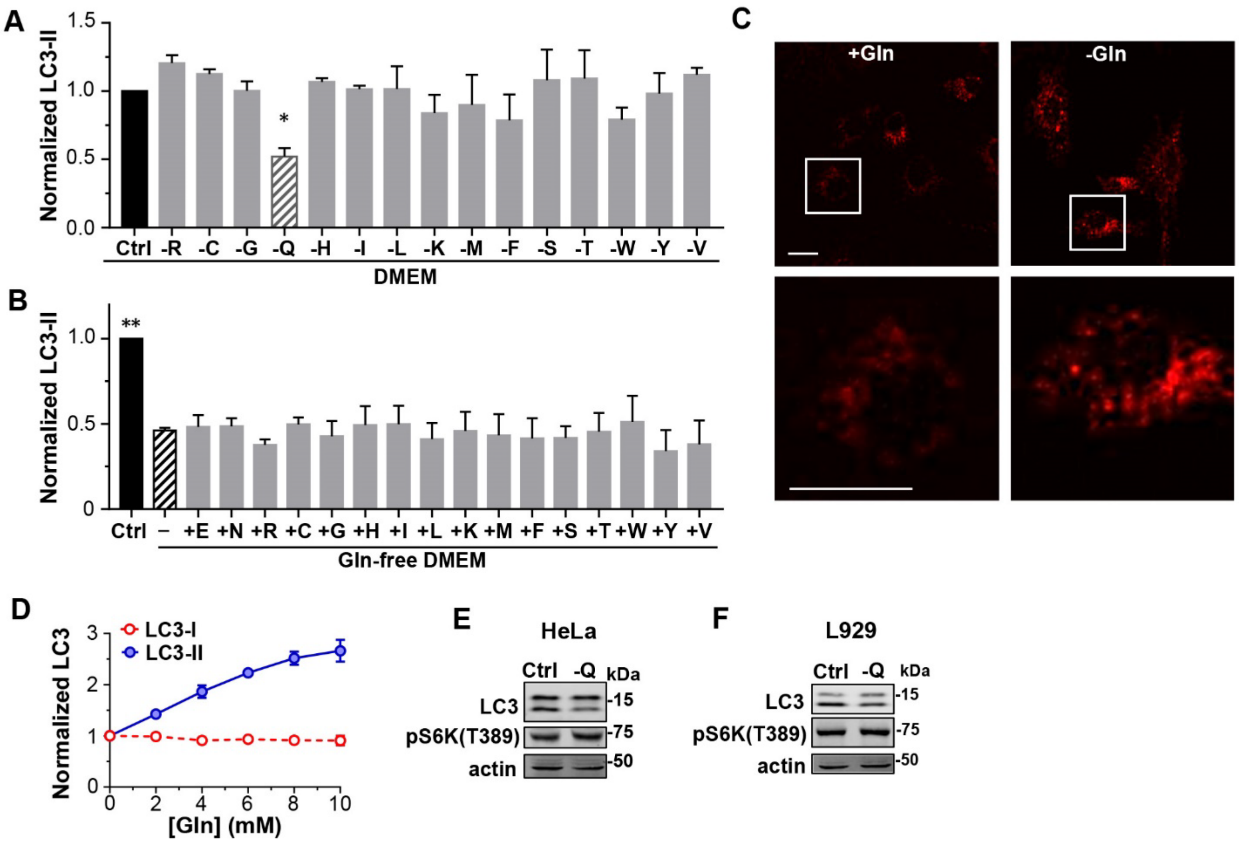

Figure S2. Glutamine withdrawal accelerates LC3-II degradation.
(**A**) Statistics of LC3-II levels normalized to that of untreated Ctrl for conditions shown in **Fig. 2A** from n = 3 experiments; LC3-II/actin ratios were used for normalization. * $p < 0.05$ *vs*. Ctrl, by one way ANOVA with Dunnett's multiple comparisons test. (**B**) Statistics of LC3-II levels normalized to that of untreated Ctrl for conditions shown in **Fig. 2B** from n = 3 experiments; LC3-II/actin ratios were used for normalization. ** $p < 0.01$ *vs*. -Gln, by one way ANOVA with Dunnett's multiple comparisons test. (**C**) Same images as that shown in **Fig. 2C**, with the red DQ-BSA fluorescence in black background and single cells in boxed areas enlarged (*lower panels*) to show lysosomes containing the degraded (dequenched) products. Scale bars, 20 μm. (**D**) Concentration response curves of LC3-II (*blue*) and LC3-I (*red*) levels to Gln in the culture media, determined from n = 3 experiments as shown in **Fig**. **2E**. Same experiments as that shown in **Fig. 2F**, with LC3-II/actin and LC3-I/actin ratios used for normalization. (**E, F**) Western blot analysis of LC3, pS6K(T389), and actin of HeLa (**E**) and L929 (**F**) cells cultured in normal medium (Ctrl) or treated for 1 hour in the Gln-free medium (-Q).

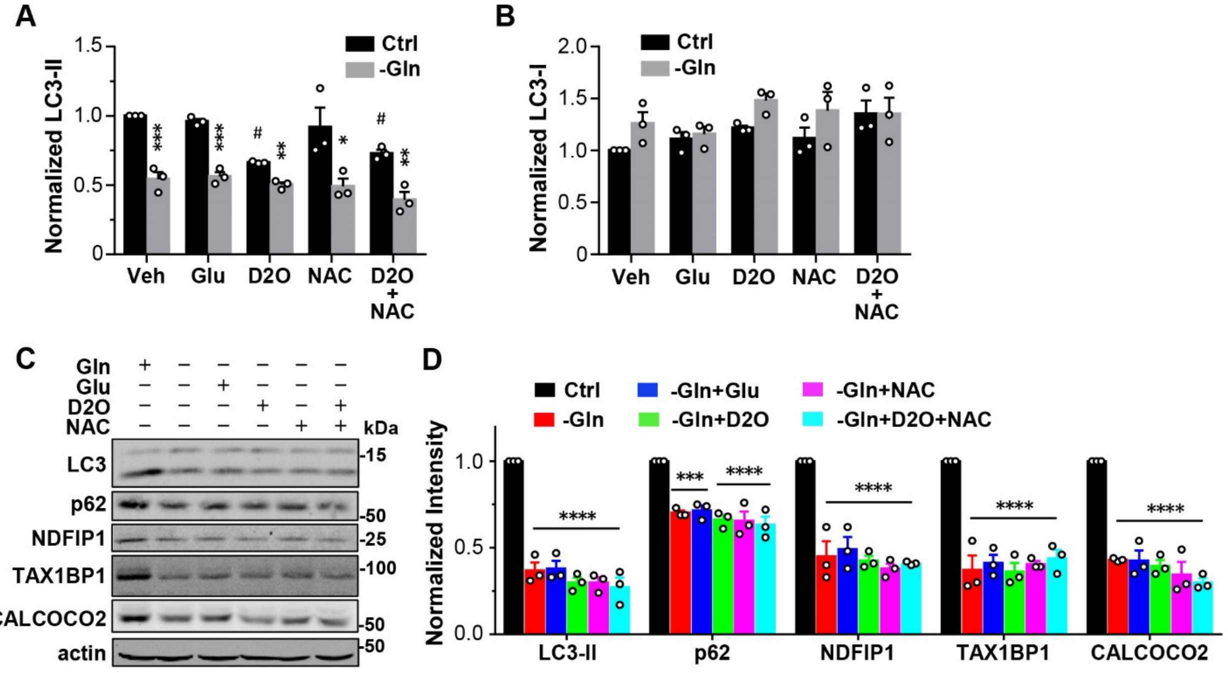

Figure S3. Glutamine regulates lysosomal degradation independently of oxidation and glutamine-driven energy metabolism. (**A, B**) Statistics of LC3-II and LC3-I levels normalized to that of untreated Ctrl for conditions shown in **Fig. 3A** from n = 3 experiments; LC3-II/actin and LC3-I/actin ratios were used for normalization. * $p < 0.05$, ** $p < 0.01$, *** $p < 0.001$ *vs*. corresponding +Gln by Grouped analyses - multiple *t* tests; $^{\#}$ $p < 0.05$ *vs*. Ctrl +Gln by one-way ANOVA with Dunnett's multiple comparisons test. (**C**) Western blot analysis of LC3, p62, NDFIP1, TAX1BP1, CALCOCO2, and actin of BJ cells cultured in normal medium (Ctrl) or Gln-free DMEM supplemented with Glu (4 mM), DM-2-OG (D2O, 6 mM), and/or NAC (6 mM) as indicated for 1 hour. (**D**) Statistics of LC3, p62, NDFIP1, TAX1BP1, CALCOCO2 levels normalized to that of untreated Ctrl for conditions shown in (**C**) from n = 3 experiments. All proteins were normalized to the loading control, actin, before the normalization to the untreated Ctrl for each experiment. *** $p < 0.001$, **** $p < 0.0001$, by one-way ANOVA with Dunnett's multiple comparisons test.

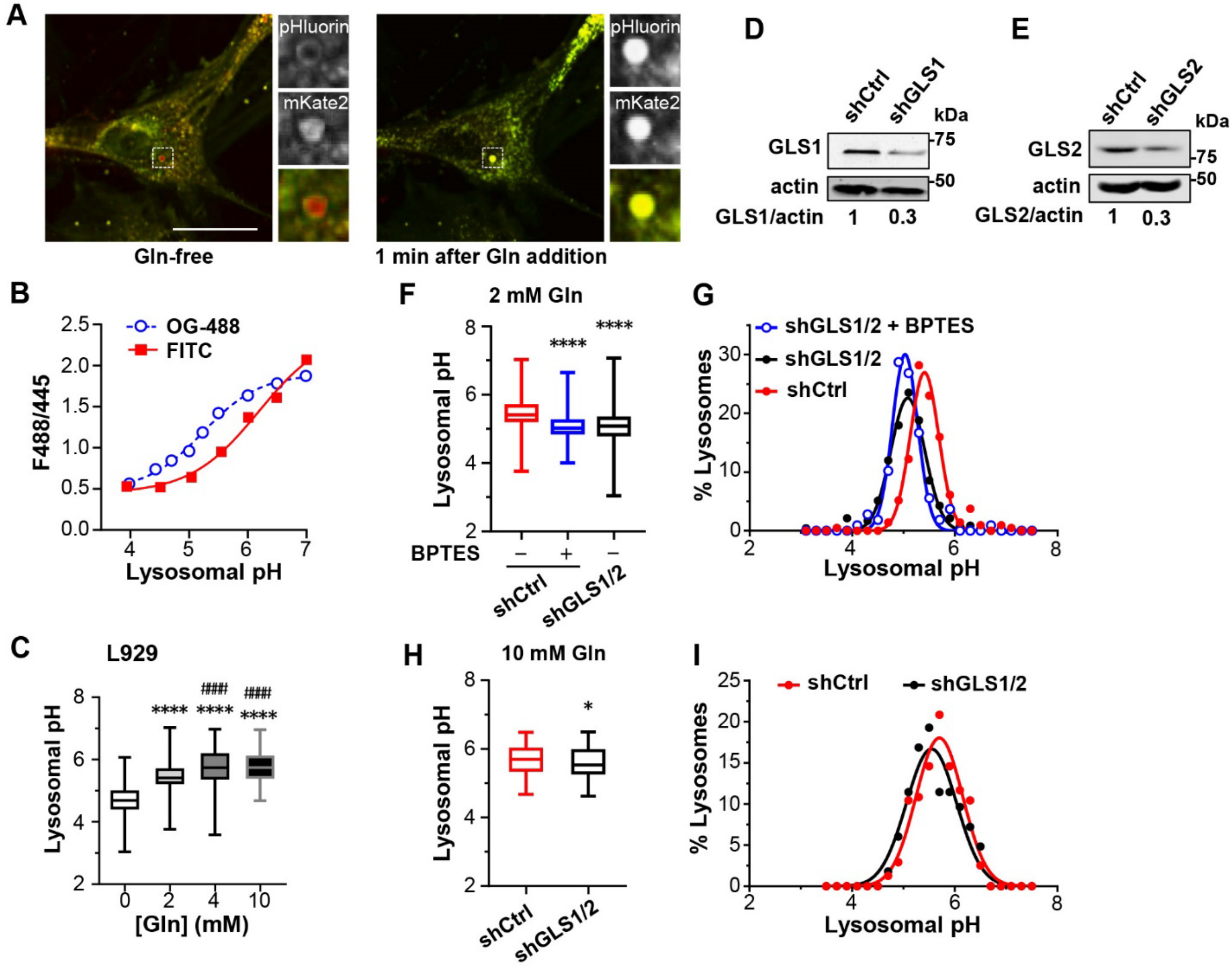

Figure S4. Effect of glutamine and glutaminases on lysosomal pH.
(A) Representative fluorescence images of MEF cells expressing PK-hLC3 maintained in the Gln-free medium *(left)* and 1 min after the addition of 4 mM Gln into the medium (*right*). Scale bar, 20 μm. Boxed areas are enlarged at the right to display the green (pHLuorin) and red (mKate2) fluorescence of a single lysosome. Colored images are merged images. (**B**) Typical pH calibration curves for lysosomes loaded with Oregon Green 488-dextran (OG-488) and FITC-dextran. (**C**) Statistics of steady-state lysosomal pH measured using FITC-dextran of L929 cells cultured in media with the indicated concentrations of Gln for 1 hour. For 0, 2, 4, and 10 mM Gln, 598, 213, 490, 261 individual lysosomes or lysosome clusters, respectively, from 30-100 cells pooled from n = 3 experiments were analyzed. **** $p < 0.0001$ vs. 0 Gln; $^{\#\#\#\#}$ $p < 0.0001$ vs. 2 mM Gln, by one way ANOVA with Tukey's multiple comparisons test. (**D, E**) Knockdown efficiency of GLS1 (**D**) and GLS2 (**E**). L929 cells were infected with lentivirus for either scrambled control shRNA (shCtrl) or shRNA that targets GLS1 (shGLS1) or GLS2 (shGLS2). Western blot analysis was performed using GLS1, GLS2, and actin antibodies. Normalized GLS1 and GLS2 values are indicated below the blots. (**F, G**) Steady-state lysosomal pH of L929 cells transfected with either shCtrl or shGLS1 plus shGLS2 (shGLS1/2). Cells were loaded with FITC-dextran and cultured in medium with 2 mM Gln for 1 hour. BPTES (20 μM) was added, as indicated, after the chase for the dextran dye and allowed to incubate with the cells for 2 hours before imaging. Shown are median values (**F**) and Gaussian distribution analyses (**G**) of pH values of 213, 108, and 234 individual lysosomes or lysosome clusters for shCtrl-BPTES, shCtrl+BPTES, and shGLS1/2-BPTES, respectively, from 30-100

cells pooled from n = 3 experiments. **** $p < 0.0001$ *vs*. shCtrl-BPTES, by one-way ANOVA with Tukey's multiple comparisons test. (**H, I**) Steady-state lysosomal pH of L929 cells transfected with either shCtrl or shGLS1/2. Cells were loaded with FITC-dextran and cultured in medium with 10 mM Gln for 1 hour. Shown are median values (**H**) and Gaussian distribution analyses (**I**) of pH values of 240 and 166 individual lysosomes or lysosome clusters for shCtrl and shGLS1/2, respectively, from 30-100 cells pooled from n = 3 experiments. * $p < 0.05$ by *t* test.

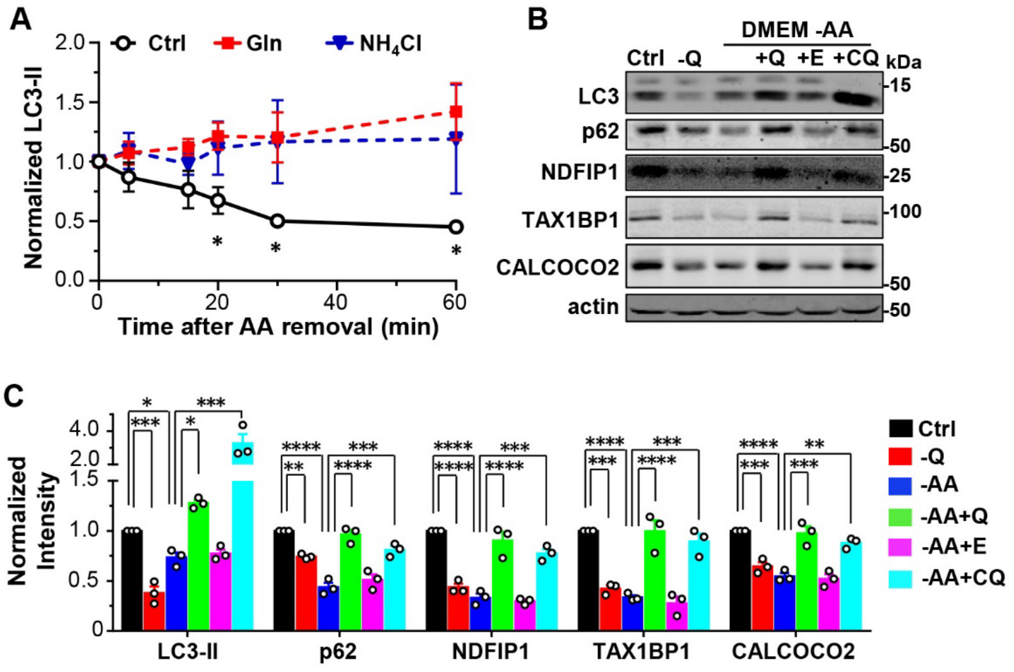

Figure S5. Glutamine supplementation prevents amino acid-starvation-induced acceleration of lysosomal degradation.

(**A**) Statistics of LC3-II levels normalized to that of unstarved cells for conditions shown in **Fig. 6A**; LC3-II/actin ratios were used for normalization. Data points are means ± SEM from n = 3 experiments. * $p < 0.05$, Ctrl vs. Gln at each of the time points by Grouped analyses - multiple t tests; $p < 0.001$ between treatment conditions, by two-way ANOVA. (**B**) Representative western blot analysis of LC3, p62, NDFIP1, TAX1BP1, CALCOCO2, and actin in BJ cells exposed to amino acid-free medium (-AA) for 1 hour. Gln (+Q, 10 mM), Glu (+E, 10 mM), and chloroquine (+CQ, 100 μM) were included in the amino acid-free medium as indicated. Normal medium (Ctrl, 4 mM Gln) and Gln-free medium (-Q) were used for positive and negative controls, respectively. (**C**) Statistics of LC3, p62, NDFIP1, TAX1BP1, and CALCOCO2 levels normalized to that of Ctrl for conditions shown in (B) from n = 3 experiments. * $p < 0.05$, ** $p < 0.01$, *** $p < 0.001$, **** $p < 0.0001$ by one-way ANOVA with Tukey's multiple comparisons test.

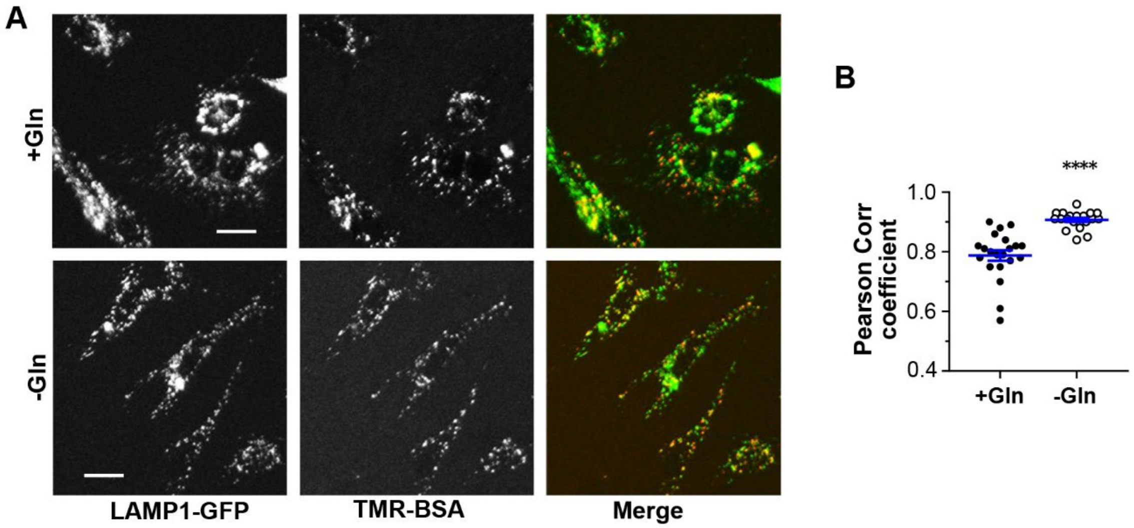

Figure S6. Glutamine deprivation enhances endocytosis of tetramethylrhodamine (TMR)-conjugated BSA.
(**A**) Representative images of TMR-BSA (*red*) taken up by MEF cells expressing LAMP1-GFP (*green*). Cells were exposed to TMR-BSA in normal culture medium (+Gln, 4 mM) or Gln-free culture medium (-Gln) for 2 hours. Images were taken immediately after washing. (**B**) Statistical analysis of the Pearson correlation coefficient between TMR-BSA and LAMP1-GFP for (A). Data points were from 6-7 coverslips with 3 areas of each coverslip. **** $p < 0.0001$ by *t* test**.**
